# Influence of support materials on the electroactive behavior, structure and gene expression of wild type and GSU1771-deficient mutant of *Geobacter sulfurreducens* biofilms

**DOI:** 10.1007/s11356-024-33612-3

**Published:** 2024-05-17

**Authors:** Luis Miguel Rodríguez-Torres, Guillermo Antonio Huerta-Miranda, Ana Luisa Martínez-García, Dalia Alejandra Mazón-Montijo, Alberto Hernández-Eligio, Margarita Miranda-Hernández, Katy Juárez

**Affiliations:** 1 Departamento de Ingeniería Celular y Biocatálisis, Instituto de Biotecnología, Universidad Nacional Autónoma de México, Av. Universidad 2001. Col. Chamilpa, 62210 Cuernavaca, Morelos México; 2 Centro de Investigación en Materiales Avanzados S. C., Subsede Monterrey, Grupo de Investigación DORA-Lab, 66628 Apodaca, N. L México; 3 Centro de Investigación e Innovación Tecnológica (CIIT), Grupo de Investigación DORA-Lab, Tecnológico Nacional de México Campus Nuevo León (TECNL), 66629 Apodaca, N. L México; 4 Investigadores Por México, CONAHCYT, Ciudad de México, México; 5 Instituto de Energías Renovables, Universidad Nacional Autónoma de México, Priv. Xochicalco, 62580 Temixco, Morelos México

**Keywords:** *Geobacter sulfurreducens*, *c-*type cytochromes, Bioelectrochemical systems (BES), Biofilm structure, Electrochemical activity, Support materials

## Abstract

*Geobacter sulfurreducens* DL1 is a metal-reducing dissimilatory bacterium frequently used to produce electricity in bioelectrochemical systems (BES). The biofilm formed on electrodes is one of the most important factors for efficient electron transfer; this is possible due to the production of type IV pili and *c-*type cytochromes that allow it to carry out extracellular electron transfer (EET) to final acceptors. In this study, we analyzed the biofilm formed on different support materials (glass, hematite (Fe_2_O_3_) on glass, fluorine-doped tin oxide (FTO) semiconductor glass, Fe_2_O_3_ on FTO, graphite, and stainless steel) by *G. sulfurreducens* DL1 (WT) and GSU1771-deficient strain mutant (Δ*gsu1771*). GSU1771 is a transcriptional regulator that controls the expression of several genes involved in electron transfer. Different approaches and experimental tests were carried out with the biofilms grown on the different support materials including structure analysis by confocal laser scanning microscopy (CLSM), characterization of electrochemical activity, and quantification of relative gene expression by RT-qPCR. The gene expression of selected genes involved in EET was analyzed, observing an overexpression of *pgcA*, *omcS*, *omcM*, and *omcF* from Δ*gsu1771* biofilms compared to those from WT, also the overexpression of the *epsH* gene, which is involved in exopolysaccharide synthesis. Although we observed that for the *Δgsu1771* mutant strain, the associated redox processes are similar to the WT strain, and more current is produced, we think that this could be associated with a higher relative expression of certain genes involved in EET and in the production of exopolysaccharides despite the chemical environment where the biofilm develops. This study supports that *G. sulfurreducens* is capable of adapting to the electrochemical environment where it grows.

**Supplementary Information:**

The online version contains supplementary material available at 10.1007/s11356-024-33612-3.

## Introduction

*Geobacter sulfurreducens* is an anaerobic δ-proteobacterium that lives in the subsurface, and it participates in the biogeochemical cycles of iron (Fe) and manganese (Mn) (Caccavo et al. 1994; Reguera and Kashefi 2019). This microorganism has generated great interest for its biotechnological applications, including the degradation of organic compounds and the reduction of various heavy metals such as uranium (U), cadmium (Cd), cobalt (Co), palladium (Pd), and technetium (Tc) (Caccavo et al. 1994; Reguera and Kashefi 2019). The *G. sulfurreducens* genome encodes more than 100 *c-*type cytochromes (Methé et al. 2003), and it expresses conductive nanowires made of type IV pili and/or self-assembled *c-*type cytochromes (Wang et al. 2019; Yalcin et al. 2020; Ye et al. 2022; Wang et al. 2022a). In nature, these two components (*c-*type cytochromes and pili) are used by *G. sulfurreducens* to extend the respiratory chain beyond the cell membranes at several distances to reduce the metallic oxides (Reguera et al. 2005).

*G. sulfurreducens* develops electroactive biofilms on electrodes to produce electricity in bioelectrochemical systems (BES) (Pant et al. 2012; Steidl et al. 2016; Tabares et al. 2019; Pinck et al. 2020). The electroactive biofilms share similarities with traditional biofilms, consisting of a complex matrix of microorganisms and extracellular polymeric substances (i.e., nucleic acids, lipids, proteins, and polysaccharides). Studies to determine the structure of the electroactive biofilms of *G. sulfurreducens* have involved confocal laser scanning microscopy (CLSM) to observe the biofilm morphology, cell viability, and maximum thickness (Wen et al. 2022). The extracellular polymer substance (EPS) is important in biofilm structure, cohesion, and anchoring redox components.

According to studies, the EET requires the participation of both *c-*type cytochromes and type IV pili to promote electron transfer reactions in both thin (> 10 μm) and thick biofilms (10–50 μm) (Bonanni et al. 2013; Steidl et al. 2016). Extensive research has focused on the multiheme *c-*type cytochromes to uncover the different pathways employed by *G. sulfurreducens* for transferring electrons from the quinone pool to low or high-potential final acceptors and to propose a mechanism for sensing the redox potential of the surrounding environment (Levar et al. 2014; Zacharoff et al. 2016; Joshi et al. 2021; Howley et al. 2023). Furthermore, the fact that this bacterium can encode more than 100 cytochromes might explain its great versatility in terms of the vast number of electron acceptors it can use. The most studied outer membrane cytochromes are the following: OmcB, OmcS, OmcZ, OmcC, OmcE, OmcF, OmcT, and PgcA (Leang et al. 2003; Kim et al. 2005; Mehta et al. 2005; Inoue et al. 2010; Qian et al. 2011; Zacharoff et al. 2017).

The genes that express these important EET proteins (*c-*type cytochromes, pili structural protein type IV, and their assembly) are regulated by transcriptional regulators whose functions have been reported for *G. sulfurreducens* (Juárez et al. 2009; Leang et al. 2009; Tremblay et al. 2011; Summers et al. 2012; Andrade et al. 2021; Hernández-Eligio et al. 2022). One of these regulators is the GSU1771 protein, identified as a member of the *Streptomyces* Antibiotic Regulatory Protein (SARP) family (Tremblay et al. 2011). Recent findings have shown that the GSU1771 protein regulates the transcription of several genes involved in the reduction of Fe(III) and the transfer of extracellular electrons to support materials (Hernández-Eligio et al. 2022; Jaramillo-Rodríguez et al. 2023). Additionally, it was found that the biofilm of the *Δgsu1771* mutant strain is thicker and with particular structures in comparison with the wild type (WT) strain and other phenotypic changes, like a delay in its growth rate in acetate-fumarate, but an increase in Fe(III) oxide reduction activity. All these features have drawn attention since the mutation also generated high electroactive biofilms (i.e., enhanced ability to transfer electrons to electrodes more efficiently than the WT strain) (Hernández-Eligio et al. 2022).

In addition to biological factors influencing biofilm formation and activity, the support material can change the properties of the electroconductive biofilms (Semenec and Franks 2015). Variations in the anode size affect the thickness of the biofilm in pure cultures of *G. sulfurreducens* (Nevin et al. 2008). Meanwhile, the chemical composition of the support material plays an important role in shaping features such as pores formation, surface morphology, roughness, and hydrophilicity (Semenec and Franks 2015). Previously, we conducted multidisciplinary research studies to describe the interaction between various support materials and the *G. sulfurreducens* WT strain; specifically, materials like fluorine-doped tin oxide (FTO) and ordinary glass were found to enhance bacterial interaction by modifying the surfaces with Fe_2_O_3_ films as observed in CLSM studies. Furthermore, our research proved that *G. sulfurreducens* exhibits different electroactive behaviors depending on the support material it interacts with. We reported that in the presence of the Fe_2_O_3_ film, the bacteria dissolved this compound instead of transferring the electrons to the current collector; in contrast, in the absence of this film, the electroactive activity of the biofilm was a typical turn-over response in the presence of sodium acetate (NaAc) (Huerta-Miranda et al. 2023).

Understanding how biofilms form on different surfaces is essential for properly developing electroactive biofilms and their use in bioelectrochemical devices. The influence of support materials on biofilm structure is also an important parameter to consider. In this study, we analyze biofilm structure and its bioelectrochemical properties using different support materials with different chemical characteristics: glass as inert non-conductive material, glass covered with iron oxides (Fe_2_O_3_-glass), and conductive materials (FTO, Fe_2_O_3_-FTO, graphite, and stainless steel). Typically, carbon-based materials, like graphite, are used for microbial anodes, but for several analyses and applications, including microscopy, a transparent support material like FTO is required (Scarabotti et al. 2021). Table 1 presents commonly used materials for studying electroactive biofilms for many applications, from energy production to biosensing and microbial electrolysis cells.

**Table 1:** Materials used as electrodes in the study of electroactive biofilms

Material	Description	Open circuit potential (V vs. SHE)	Microorganisms	References
Glass	Hard, brittle, transparent, and amorphous inorganic material	N/A	*Geobacter sulfurreducens*	Yi et al. 2009; Cologgi et al. 2014; Richter et al. 2017; Huerta-Miranda et al. 2019; Huerta-Miranda et al. 2023; Jaramillo-Rodríguez et al. 2023
			*Shewanella oneidensis* MR-1	Thormann et al. 2004; Chao et al. 2013
ITO	Transparent semiconductor indium tin oxide	0.15 – 0.2^a^	*Geobacter sulfurreducens*	Jain et al. 2011; Matsuda et al. 2011; Robuschi et al. 2017; Ren et al. 2021; Frühauf et al. 2022
			*Azospirillum humicireducens*	Chen et al. 2023; Wu et al. 2023
			*Shewanella oneidensis* MR-1	Kuo et al. 2024
			Microbial consortium	Saavedra et al. 2023
FTO	Transparent semiconductor of fluorine-doped tin oxide	0.39—0.43^b^	*Geobacter sulfurreducens*	Huerta-Miranda et al. 2019; Zhang et al. 2021; Hernández-Eligio et al. 2022; Neu et al. 2022; Huerta-Miranda et al. 2023
			*Chlorella vulgaris*	Thorne et al. 2011; Saifuddin et al. 2022
			Microbial consortium	Heijne et al. 2018; Molenaar et al. 2018; Ueoka et al. 2018; Pereira et al. 2022; Sridharan et al. 2022
Fe_2_O_3_	A layer of hematite on different type of materials	0.2—0.3^c^	*Geobacter species*	Kato et al. 2013; Li et al. 2014; Huerta Miranda et al. 2023
			*Shewanella oneidensis* MR-1	Meitl et al. 2009; Johs et al. 2010; Qian et al. 2014; Zhou et al. 2015; Gao et al. 2019
			*Shewanella putrefaciens* CN-32	Hu et al. 2020
			*Pseudomonas aeruginosa*	Ren et al. 2017
			Microbial consortium	Liang et al. 2016; Wen et al. 2022
Graphite plate	A brittle, black, semiconducting form of carbon	0.1—0.55^d^	*Geobacter sulfurreducens*	Bond and Lovley 2003; Marsili et al. 2008; Katuri et al. 2012; Jana et al. 2014; Huerta-Miranda et al. 2023; Jaramillo-Rodríguez et al. 2023
			*Shewanella oneidensis* MR-1	Matsumoto et al. 2021
			*Listeria monocytogenes*	Light et al. 2018
			*Thermincola ferriacetica*	Lusk et al. 2016; Faustino et al. 2021
			Microbial consortium	Liu et al. 2007; Tavakolian et al. 2020
Carbon paper	Composite material of carbon fiber and carbon	0.33^e^	*Escherichia coli*	Zhao et al. 2012
			*Acetivibrio thermocellus* DSM 1313	Yan and Zhu 2023
			*Thermoanaerobacterium thermosaccharolyticum* MJ2	Yan et al. 2023
			Microbial consortium	Min and Logan 2004; Uria et al. 2017
Stainless steel	Steel alloy that contains other metals in different quantities, such as chromium, molybdenum, nickel and tungsten	0.15—0.2^f^	*Geobacter sulfurreducens*	Dumas et al. 2008; Tang et al. 2019; Tang et al. 2021
			*Geobacter metallireducens*	Tang et al. 2021
			Microbial consortium	Pocaznoi et al. 2012; Ledezma et al. 2015; Pu et al. 2018
Gold	Highly conductive and chemically inert metal electrode	0.17—0.21^ g^	*Geobacter sulfurreducens*	Richter et al. 2008; Liu et al. 2011; Vargas et al. 2013; Kuzume et al. 2013; Maestro et al. 2014; Zhang et al. 2016; Champigneux et al. 2018; Füeg et al. 2019; Scarabotti et al. 2021
			*Shewanella oneidensis* MR-1	Kane et al. 2013
			*Escherichia coli*	Borghol et al. 2010

^a^Matveeva 2005; ^b^Korjenic and Raja 2019; ^c^Shimizu et al. 2012; ^d^Renslow et al. 2011; ^e^Alvarado-Ávila et al. 2020; ^f^Tang et al. 2021; ^g^Pinto et al. 2019

Additionally, our research involved a combination of electrochemical techniques and complemented them with CLSM to identify the optimal material for generating thick and homogeneous biofilms and to produce high currents in catalytic conditions in the oxidation of sodium acetate. Additionally, we conducted a comparative analysis of gene expression between the *Δgsu1771* strain and the WT strain across all tested materials. Our results revealed that this mutation significantly enhances the differential expression of genes involved in biofilm formation and bioenergy production, notably in genes related to *c-*type cytochromes like PgcA, OmcS, or OmcZ and others that have been less frequently reported like OmcF or OmcM.

## Materials and methods

### Bacterial strains and culture conditions

This study compares two strains of *G. sulfurreducens* DL1 (WT) and the *Δgsu1771* mutant strain (Hernández-Eligio et al. 2022). Both bacteria strains were routinely cultivated under anaerobic conditions in NBAF medium with 30 mM sodium acetate (NaAc) as the electron donor and 40 mM sodium fumarate as the electron acceptor (Coppi et al. 2001). We employed six different materials as support and/or electrodes: glass, glass covered with Fe(III) oxide (Fe_2_O_3_-glass) (Mazón-Montijo et al. 2020), fluorine-doped tin oxide (FTO), FTO covered with Fe(III) oxide (Fe_2_O_3_-FTO) (Huerta-Miranda et al. 2023), graphite plate, and stainless steel. The cultures were incubated for 48 h in hermetically sealed test tubes at 25 °C, without agitation. Before conducting any study, the strains underwent an “adaptation” process following established protocols in earlier reports (Hernández-Eligio et al. 2022).

### Observation of the biofilm structure by CLSM

The dye mixture of the “LIVE/DEAD Bacterial Viability Kit” was prepared based on the instructions given in previous reports (Hernández-Eligio et al. 2022). The image analysis and biofilm parameters were performed using Comstat2 (version 2.1) and Fiji (version 2.9.0) software (Heydorn et al. 2000; Schindelin et al. 2012).

### Electrochemical methods

All electrochemical studies were conducted in a conventional three-electrode cell with Ag/AgCl as the reference electrode; 0.199 V vs. standard hydrogen electrode (SHE), all the potentials reported herein refer to the SHE. The counter electrode was a platinum plate, and the biofilms grown on the different support materials were the working electrodes. At 48 h of incubation time (except for graphite, in which the selected time was 96 h), the biofilm/electrodes were carefully removed from the sealed test tubes and placed inside the electrochemical cell. The electrochemical cell consisted of a hermetic glass chamber bubbled with a gas mixture of N_2_ to CO_2_ (80:20). The basal medium (BM) (Hernández-Eligio et al. 2020) was used as the electrolytic solution. Open circuit potential (OCP), cyclic voltammetry (CV), and square wave voltammetry (SWV) were the electrochemical techniques used in this study. The OCP was measured for 10 min. The CV in non-catalytic and catalytic conditions (adding 20 mM NaAc) was performed in different potential windows depending on the support material (at 0.01 V/s scan rate): from − 0.93 to 0.48 V in FTO, from − 0.55 to 1.0 V in Fe_2_O_3_-FTO, − 0.74 to 0.99 V in graphite, and − 0.64 to 0.72 V in stainless steel. The SWV was performed at a step potential of 0.001 V, 0.01 V modulation amplitude, and a frequency of 30 Hz. The scan started from negative to positive potentials in the same potential windows as CV.

### Gene expression and quantitative reverse transcription PCR (RT-qPCR)

Total RNA was extracted from *G. sulfurreducens* biofilms grown on all support materials in NBAF medium at 25 °C at 48 h. mRNA extraction was carried out using the RNeasy Mini Kit (Qiagen), and then residual DNA was removed using DNase I (Thermo Scientific). Complementary DNA (cDNA) synthesis was performed using the RevertAid H Minus First Strand cDNA Synthesis kit (Thermo Scientific) and the specific reverse oligonucleotides (Supplementary Table S1). Afterward, qPCR was performed using the Maxima SYBR Green/ROX qPCR Master Mix (Thermo Scientific) and specific oligonucleotides (Supplementary Table S1) using a Rotor-Gene^R^ Q (Qiagen). The gene-specific oligonucleotides used for RT-qPCR are summarized in Supplementary Table S1. *recA* and *gsu2822* were used as internal gene standards for PCR amplification. Normalized relative expression fold changes were quantified via the 2^−ΔΔCT^ method using the Rotor-Gene Q Series Software program. All experiments were conducted in triplicate, and the results were averaged.

## Results and discussion

### Electrode materials analyzed and biofilm structure analysis by CLSM

**Fig. 1:**
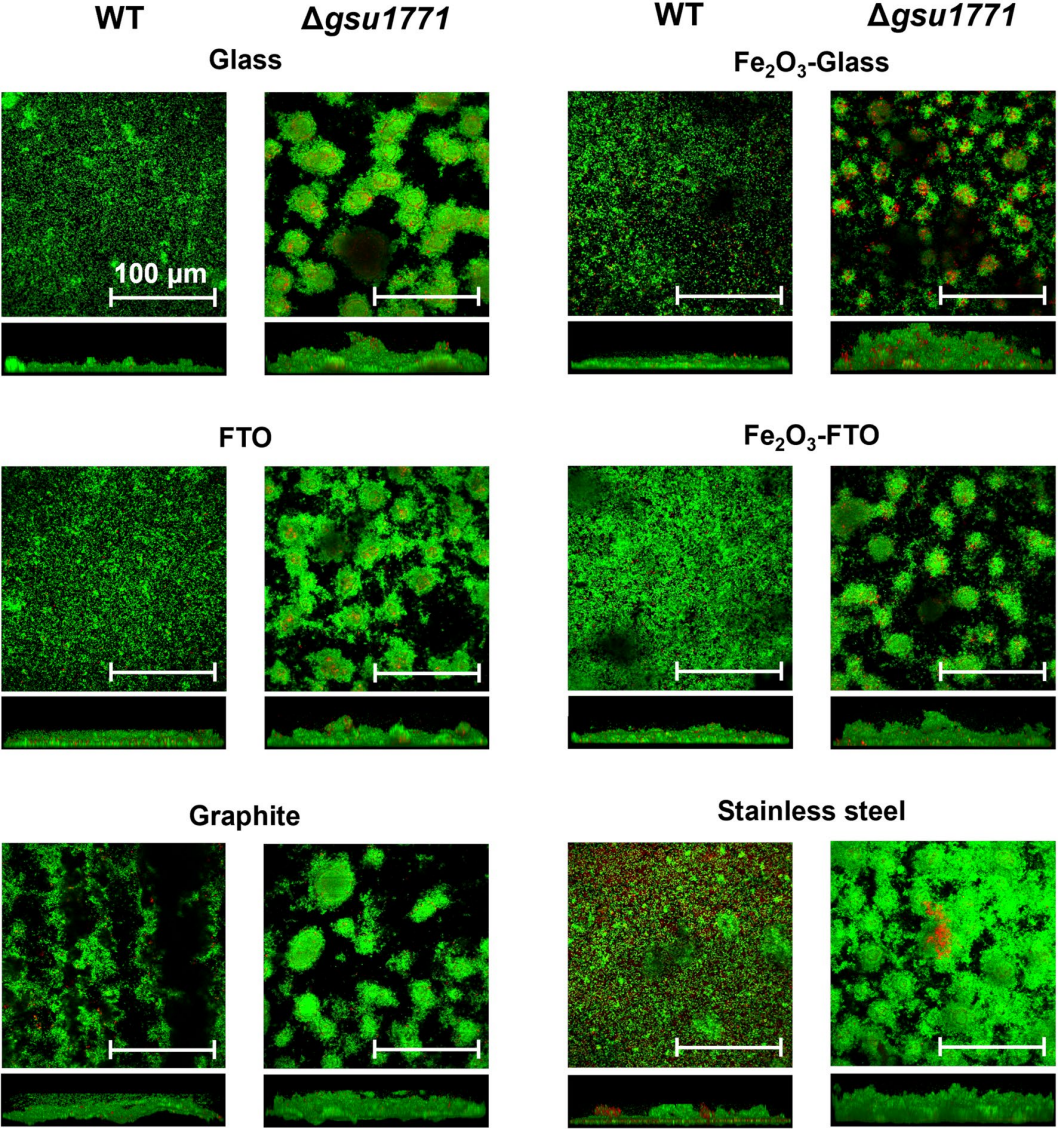
CLSM analysis of DL1 (WT) and Δ*gsu1771* biofilms formed on different supports (top and lateral views). The WT and Δ*gsu1771 G. sulfurreducens* biofilms were grown for 48 h at 25 °C. The biofilms were stained with the LIVE/DEAD bacterial viability kit. Living cells stain green, and dead cells stain red. The white line indicates a scale of 100 μm. The materials used to grow the biofilms are indicated at the top of each panel

In this study, we analyze the biofilm structure and its bioelectrochemical properties using different electrode materials with different chemical characteristics: glass as inert non-conductive material, glass covered with iron oxides (Fe_2_O_3_-glass), and conductive materials (FTO, Fe_2_O_3_-FTO, graphite, and stainless steel). Figure 1 shows the three-dimensional structure of the biofilms by CLSM on all the previously mentioned supports: glass, Fe_2_O_3_-glass, FTO, Fe_2_O_3_-FTO, graphite, and stainless steel. The parameters obtained from the image analysis are reported in Table 2. The biofilms formed by the WT strain show several differences in dependence on the support material: on glass, the biofilm is distributed homogeneously on the surface, and its viability was more than 90%; however, the biofilm thickness was the lowest. When the glass was covered with the hematite film (Fe_2_O_3_-glass), the viability and homogeneity of the WT biofilm did not significantly change, but it was 1.5-fold thicker than when it grew on bare glass. We previously reported that by using Fe_2_O_3_ as an electron acceptor, which closely resembles the natural environments of *G. sulfurreducens*, the formation of thicker biofilms compared to the unmodified bare surface is promoted (Huerta-Miranda et al. 2023).

**Table 2:** Biofilm parameters of the CLSM image analysis

	Material	Strain*	
		WT	*Δgsu1771*
Thickness (μm)	Glass	13.2 ± 0.8	58.7 ± 6.2
	Fe_2_O_3-_glass	20.2 ± 2.2	68.5 ± 18.5
	FTO	22.7 ± 3.4	39.2 ± 6.1
	Fe_2_O_3_-FTO	21.0 ± 2.0	45.0 ± 0.5
	Graphite	39.3 ± 2.2	39.3 ± 6.4
	Stainless steel	24.8 ± 2.1	44.2 ± 4.7
Cell viability (%)	Glass	97.4 ± 0.8	79.5 ± 1.4
	Fe_2_O_3-_glass	91.0 ± 3.5	78.9 ± 0.3
	FTO	88.7 ± 2.3	92.6 ± 0.2
	Fe_2_O_3_-FTO	86.7 ± 4.3	84.1 ± 2.4
	Graphite	93.7 ± 0.4	92.1 ± 0.7
	Stainless steel	74.3 ± 0.7	92.9 ± 1.6

*The results are the average values of *n* > 2 samples and their standard error ( ±)

The *Δgsu1771* strain forms a non-continuous biofilm (areas with regular agglomerates of cells) considerably thicker than the WT strain on both bare glass and Fe_2_O_3_-glass (4.4- and 3.4-fold, respectively), also observing the effect of greater thickness in the glass modified with Fe_2_O_3_ (a thickness increment of 1.2-fold compared with bare glass). The increment in thickness affected the viability of the biofilms, as other authors mentioned (Islam et al. 2017; Zhuang et al. 2022). The mutant biofilm exhibits patterns (see top views) related to column-like structures with channels, which are not present in the WT strain.

The results with FTO showed that the WT strain formed a homogeneous biofilm with viability > 80%. When FTO was modified with the Fe_2_O_3_ film, the viability and thickness were conserved but high cell accumulation was observed. The *Δgsu1771* biofilm on FTO shows the same localized growth observed on the glass; we previously reported this behavior in bare FTO support electrodes (Hernández-Eligio et al. 2022). Compared to the WT biofilm, the *Δgsu1771* strain forms 1.7-fold thicker biofilms and 2.1-fold thicker biofilms on bare FTO and Fe_2_O_3_-FTO, respectively. These results suggest that the *Δgsu1771* biofilm shows more sensitivity to the modification with Fe_2_O_3_ than the WT biofilm since the thickness and the cell viability are higher. Our research group reported the interaction between *G. sulfurreducens* and the Fe_2_O_3_ (Huerta-Miranda et al. 2023) which agrees with other reports that highlight this iron oxide as a promoter of biofilm formation in many microorganisms (Zhou et al. 2015; Ren et al. 2017; Wen et al. 2022).

On graphite, the WT strain produces non-continuous biofilms (many black areas in the confocal images), which are also observed in the side view images; structurally, graphite is not a flat material like glass or FTO. Also, the porosity could be responsible for the heterogeneous distribution of this biofilm. It should be noted that the *Δgsu1771* strain formed a biofilm with similar characteristics to the other support materials. On the other hand, the thickness and viability values of both biofilms were close to those obtained on other supports; in fact, on graphite, the greatest thickness was obtained for WT biofilm.

Finally, the biofilms formed on stainless steel were homogeneously distributed. The WT strain developed a biofilm with similar thickness values as those formed on Fe_2_O_3_ but with very low viability (the lowest among all the WT biofilms). The mutant strain reaches thicknesses similar to those on Fe_2_O_3_, but unlike WT, the percentage of viability is the highest of all the tested support materials with this strain. Other authors have observed abundant biofilm formation of *G. sulfurreducens* over stainless steel (Dumas et al. 2008; Tang et al. 2021). In the literature, it has been reported that iron-reducing bacteria such as *G. sulfurreducens* promote the corrosion of iron oxides in the initial stages of biofilm formation, but in more biofilm-mature stages, it promotes the protection of the material, which is strongly influenced by environmental factors (Herrera and Videla 2009; Jin and Guan 2014). Our results indicate that the corrosion of the stainless steel is the resultant phenomenon after 40 days of incubation. Supplementary Fig S1 shows the SEM images of stainless steel electrodes after 40 days of incubation in NBAF media. The surface wear of stainless steel is evident in the biologically treated electrodes compared to the abiotic control. In addition, energy dispersive X-ray analysis (EDX) shows a decrease and an increase in the percentage of iron (Fe) and oxygen (O) atoms, respectively, relative to the total number of atoms on the surface (see Fig. S1).

These results show that the WT strain is more susceptible to changes in the support material than the Δ*gsu1771* mutant. Notably, the main reason causing the Δ*gsu1771* strain to form these structures is a current topic in our research group. Additionally, it is also important to find the molecular basis causing this mutant strain to develop biofilms with similar characteristics despite the used support material. In a recent study, through transcriptome analysis by RNA-seq, we reported that 467 genes changed their relative expression in Δ*gsu1771* biofilms grown on glass supports, compared to WT biofilms. Among the upregulated genes in the Δ*gsu1771* strain, we found those related to the synthesis of exopolysaccharides, which could explain the increased thicknesses of biofilms in this strain compared to WT (Jaramillo-Rodríguez et al. 2023).

### Electrochemical characterization of electroactive biofilms

The electrochemical techniques performed to analyze the electroconductive biofilms of *G. sulfurreducens* in a bioelectrochemical system were the open circuit potential (OCP), cyclic voltammetry (CV), and square wave voltammetry (SWV). These techniques describe the electrochemical environment at the support materials/biofilm interface, the electroactivity of the biofilms, and the potentials associated with the *c-*type cytochromes in contact with the electrode, which perform the EET reactions (Hernández-Eligio et al. 2022). It is worth noting that the electrochemical studies could only be performed on the conductive supports FTO, Fe_2_O_3_-FTO, graphite, and stainless steel; in the case of glass and Fe_2_O_3_-glass, these studies were not possible due to the non-conductive nature of these materials. Furthermore, it is important to mention that during the incubation period with the graphite electrodes, we detected a delay in the growth time of both strains, which was reflected in the electrochemical responses of the biofilms. Supplementary Fig. S2 shows the electrochemical responses of WT and *Δgsu1771* biofilms on graphite electrodes at different incubation times. The mutant *Δgsu1771* had the most significant change in the electrochemical response at 96 h. Based on these results, we selected 96 h for incubation time since the biofilm EET overcame the carbon material’s capacitance. It is well known that carbon materials have been widely used in several bioelectrochemical applications, where carbon materials are the material of choice as anode due to their biocompatibility and chemical and microbiological stability (Baudler et al. 2015; Schröder et al. 2015). In addition, carbon materials offer advantages such as low cost, wide potential window, and stability in a broad potential window (McCreery 2008). Depending on how carbon materials are manufactured, they can present different physicochemical properties that can influence the electrochemical responses of the biofilms. Carbon materials present a diverse surface area where the porosity can vary enormously. For example, a large surface area means a large adhesion surface for the biofilm; however, this propriety can lead to capacitive current increases, so in this case, the faradaic electrochemical responses of the biofilm can decrease (Heijne et al. 2018).

**Fig. 2:**
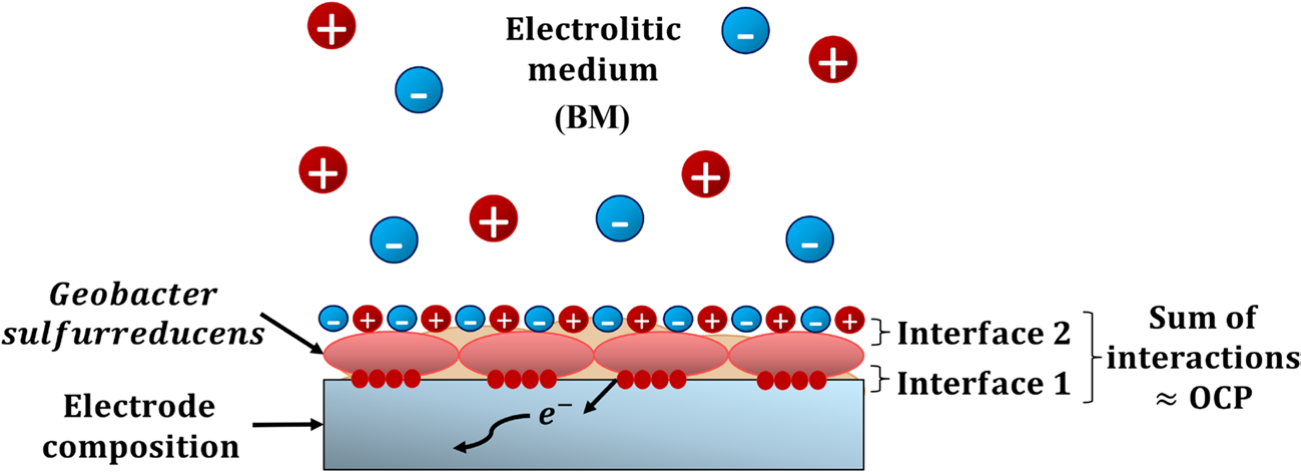
Representation of the physicochemical interactions involved in the OCP values of WT and Δ*gsu1771 G. sulfurreducens* biofilms developed on the support materials

The measurement of the OCP informs about the interfaces of a complex system like biofilms formed on electrodes. In microbial fuel cells (MFCs), the development of the OCP of a cathode can be explained as the transport of electrons from the electrode to soluble electrochemically active chemical species (Renslow et al. 2011). In the case of anodes, the OCP of an electrode with *G. sulfurreducens* biofilm shifts towards negative values due to the reduction of bacterial electroactive molecules at the biofilm/electrode interface, so the more negative, the more reductive capacity the biofilm will have (see Fig. 2) (Schrott et al. 2019; Hernández-Eligio et al. 2022). Electrochemical systems generally exhibit unique OCP values, which are determined by the physical and chemical interactions between the electrode materials, the biofilm, and the electrolytic medium. Any perturbation in the system will cause a change in the OCP, indicating a change at the electrode/biofilm interface (Schrott et al. 2019). Ion adsorption, microorganism desorption, biofilm detachment, or electrochemical reactions could cause changes in this parameter (Huerta-Miranda et al. 2019; Yates et al. 2018). In the results presented herein, we did not impose an external electrical potential during biofilm development; additionally, the electrochemical measurements were performed under conditions different from those of the culture media. As there was no external influence on biofilm development, all of our results are only attributed to the expressed phenotype of each strain.

**Fig. 3:**
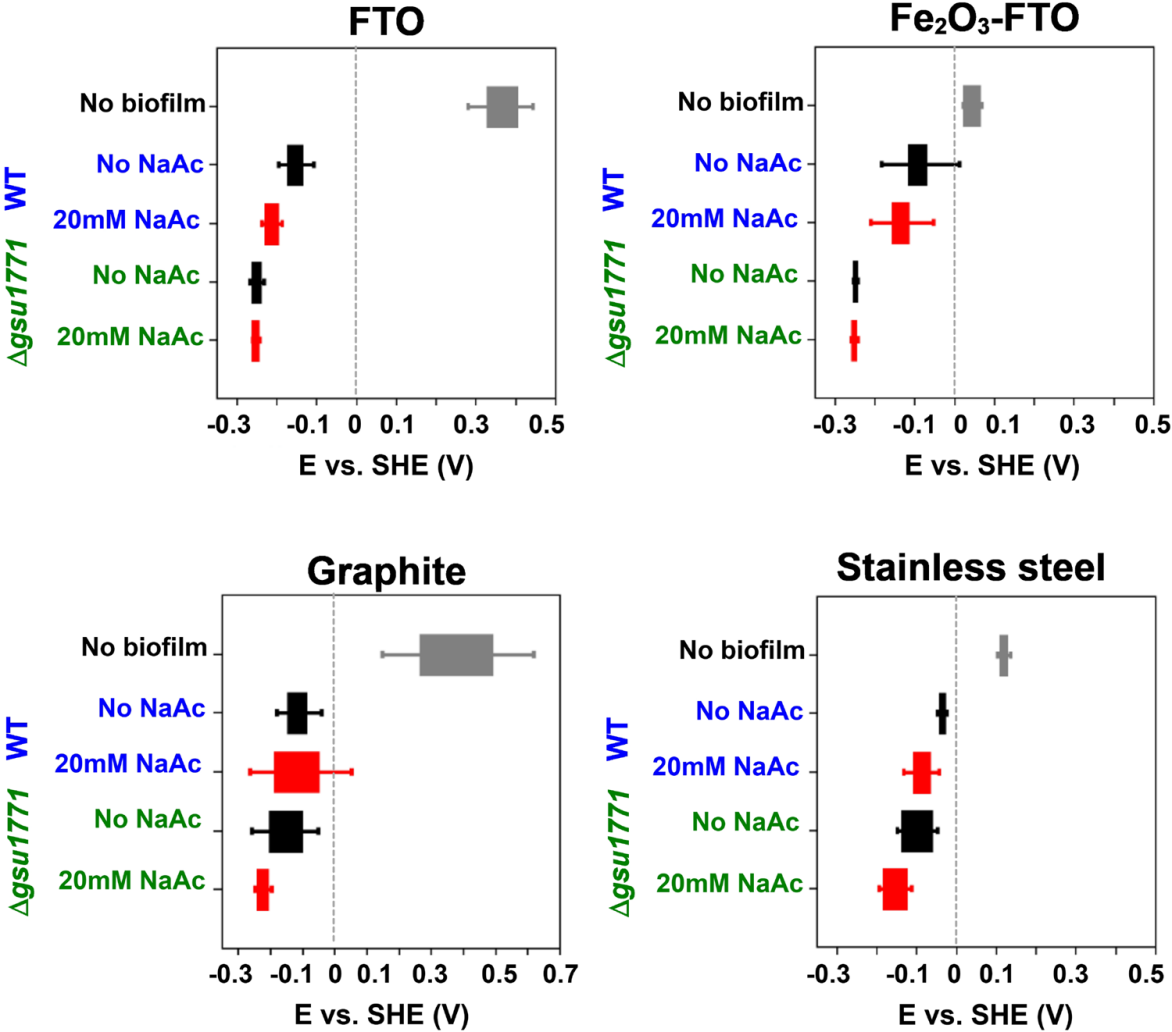
Open circuit potential (OCP) distribution of WT and Δ*gsu1771* biofilms on FTO (48 h), Fe_2_O_3_-FTO (48 h), graphite (96 h), and stainless steel (48 h). In all panels, the gray bars represent the support material (without biofilms), the black bars represent the electrochemical system without the addition of NaAc, and the red bars represent the electrochemical system after the addition of NaAc

Figure 3 shows the average OCP value after a 10-min measurement of biofilms from both strains (WT and Δ*gsu1771*) in all the support materials. The electrodes without biofilm show different potential values among each other. Graphite has the widest range and the most positive OCP value (0.3–0.5 V). The Fe_2_O_3_-FTO has the most negative potential (approx. 0.05 V). The stainless steel has an OCP of around 0.15 V. Furthermore, this could be related to the results observed in CLSM analysis, we observed that the WT strain forms thicker and homogeneous biofilms in the most negative materials (FTO, Fe_2_O_3_-FTO, stainless steel) and heterogeneous and irregular biofilms in the most positive material (graphite).

The WT biofilm grown on FTO has OCP values between − 0.1 and − 0.2 V without the addition of NaAc to the electrolyte, but in the presence of this organic molecule, the OCP shifted to more negative values; this indicates that the biofilms are electroactive towards acetate oxidation and the subsequent electron transfer to the electrode. Our group has reported this behavior previously, and we suggest that a negative shift in the OCP value indicates that the biofilms are in catalytic conditions (Hernández-Eligio et al. 2022). The Δ*gsu1771* strain has more negative OCP values than WT in the absence and presence of acetate; we reported similar changes in this condition before (Hernández-Eligio et al. 2022). Biofilms developed on Fe_2_O_3_-FTO presented OCP values that shifted to more negative potentials than the empty electrode. This behavior could mean that the Fe_2_O_3_ film promotes reducing environments, which is expected because *G. sulfurreducens* is a Fe(III) reducing microorganism.

The OCP values of the WT biofilm on graphite range from − 0.03 to − 0.17 V without NaAc and slightly change to negative potentials when this molecule is added. The *Δgsu1771* biofilm has more tendency to negative values than the WT biofilm. The wide range of OCP values with graphite with and without biofilms could result from the material’s physical characteristics. Graphite, unlike flat materials such as FTO, may have limitations for the non-homogeneous distribution of the electrolyte and the biofilm, so the formation and stabilization of the interfaces; thus, the OPC could take a long time, resulting in the broad dispersion of OCP values (Madjarov et al. 2017).

In stainless steel, the OCP values of the biofilm formed by the WT strain range between − 0.03 and − 0.06 V without NaAc and show a slight change when this compound is added. Meanwhile, the Δ*gsu1771* biofilm has more negative OCP values than the WT biofilm; in the presence of acetate, the OCP becomes slightly more negative. The OCP values of the WT biofilms are more variable in each support material used than the OCP values recorded from the Δ*gsu1771* biofilm, which remained in ranges of − 0.3 V independent of each material used. These results suggest that WT biofilms are more susceptible to the material surface than Δ*gsu1771* biofilms.

**Fig. 4:**
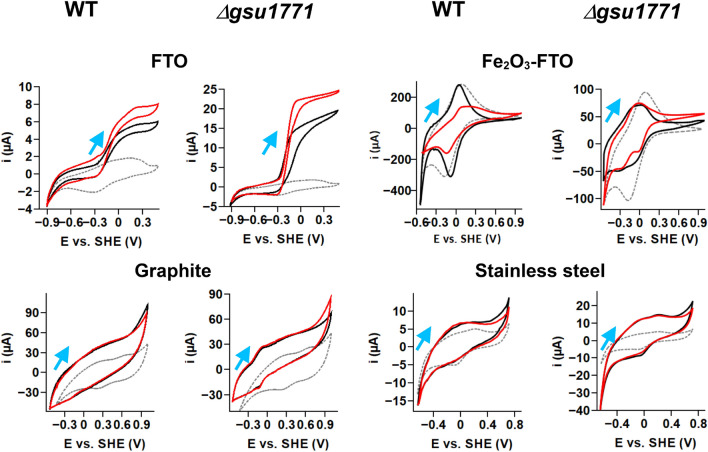
Cyclic voltammetry (CV) of *G. sulfurreducens* WT and Δ*gsu1771* biofilms on FTO (48 h), Fe_2_O_3_-FTO (48 h), graphite (96 h), and stainless steel (48 h) at 0.01 V/s scan rate. The gray-dashed lines represent the support material (without biofilms), the black lines represent the electrochemical systemwithout NaAc, and the red lines represent the electrochemical system after the addition of NaAc. The blue arrows indicate the direction of the potential scan

Figure 4 shows the CV responses of the biofilms in all the tested support materials with and without the addition of NaAc. Biofilms from both strains showed an s-shaped voltammogram in the FTO, which indicates electroactivity due to acetate metabolism (red line). The behavior of these strains with this support material is consistent and expected in terms of the potential at the inflection point of the curves reported by our work group previously (≈0.18 V) (Hernández-Eligio et al. 2022).

The Fe_2_O_3_-FTO presents the highest currents and are very similar to those without biofilm, indicating that the observed process is the Fe^3+^/Fe^2+^ redox pair of hematite. We observe a decrease in the peak currents in the presence of acetate; this indicates that the concentration of the electroactive species responsible for that redox response is decreasing at the electrode/biofilm interface. We have previously reported and confirmed the degradation of the Fe_2_O_3_ film on the Fe_2_O_3_/FTO support electrodes. By X-ray diffraction (XRD), we observed that some of the characteristic peaks of the hematite phase (Fe_2_O_3_) decreased in intensity; simultaneously, the tin oxide (SnO_2_) planes gained intensity. Also, there were no additional diffraction peaks in the electrodes in contact with the bacteria, suggesting that the redox reactions do not involve the conversion of Fe_2_O_3_ into any other iron oxide. Furthermore, using ferrozine assay, we quantified the total Fe(II) in the NBAF medium of biologically treated Fe_2_O_3_/FTO electrodes. The results indicated that *G. sulfurreducens* dissolved the Fe_2_O_3_ film and formed an unknown compound, which was released into the NBAF culture medium. Electrochemically, we observed the current decrease of Fe^3+^/Fe^2+^ redox pair of hematite due to concentration decrease as the incubation days passed. Detailed studies about these results are reported in Huerta-Miranda et al. (2023).

In a CV experiment, the measured current is usually the sum of a faradaic current (associated with the redox transformations of molecules close to the electrode) and a capacitive current, which is not involved in electron transfer. The capacitive current is a consequence of the variation of the electrode potential (Léger 2013). In our experiments, we observed that graphite is a material with high capacitance (Heijne et al. 2018); this is the cause of the low faradaic currents of the biofilms. The WT biofilms voltammograms show no clear redox processes, and when we add NaAc, there is no difference between the voltammograms. On the other hand, the *Δgsu1771* biofilm presents a reversible redox peak around − 0.1 V, but the voltammogram did not change in the presence of NaAc.

In industrial applications, stainless steel is selected over other materials because of its properties, cheaper cost, and availability in the market. Particularly, stainless steel 316 (like the one presented herein) is a boiler-grade steel used in pressure vessels. This grade has high corrosion resistance and can be operated at elevated temperatures. The chemical composition of stainless steel 316 has been reported in the literature (Bharath et al. 2014; Tang et al. 2021).

The electrochemical responses of stainless steel are influenced by its composition; the observed CV response is typical of stainless steel 316 in the voltage range of − 0.7 to 0.7 V, and it is associated with the formation of Fe(II), Fe(III), Cr(III), and Cr(VI) oxides (Minnikanti et al. 2010). The observed electrochemical responses of the stainless steel with biofilms are very similar to those without biofilm, indicating that the biofilm does not transfer electrons to the material in a similar process as FTO. However, the CSLM images show a high level of colonization and cell viability from both biofilms; thus, the stainless steel/microorganisms interaction is favorable for biofilm formation. Corrosion of the electrode explains our results obtained in this material. The corrosion of stainless steel by this microorganism was proven in culture conditions in the literature; those results suggested that *G. sulfurreducens* relied on direct electron uptake when grown on stainless steel, and it was found that the *c-*type cytochrome OmcS is important to carry out this corrosion process on this material (Tang et al. 2021). The corrosion phenomenon in stainless steel starts with the oxidation of Fe^0^ on the surface of this material, Fe^0^ is oxidized by *G. sulfurreducens* through direct metal-microorganism electron transfer, giving Fe^2+^, and part of this process generates H^+^, which is consumed by hydrogenases catalytic activity (Tang et al. 2019, 2021). Thus, *G. sulfurreducens* uses stainless steel as a cathode, so it presents the phenomenon of microbiologically influenced corrosion (MIC) in which the biofilm interacts with the iron of the stainless steel (Puentes-Cala et al. 2022; Wang et al. 2022b). This material is important for its potential use in METs (Pocaznoi et al. 2012), so to know how *G. sulfurreducens* interacts with stainless steel in our working conditions, we complemented the CLSM, SEM, EDX, and electrochemical results on this support material with the relative expression of selected genes between WT and the *Δgsu1771* mutant (see next section).

In the context of MFC, according to some authors, it is crucial for an electrode to have a high available surface area for efficient EET and biocompatibility to rely on direct contact with electroactive microorganisms like *G. sulfurreducens* (Beuth et al. 2020; Frühauf et al. 2022). However, according to our results, having good colonization on an electrode surface is not sufficient to guarantee the production of usable current coming from the microbial metabolism of *G. sulfurreducens*; the FTO is the support material that promoted electroactive biofilm development and facilitated the EET reaction towards the oxidation of acetate in both biofilms WT and *Δgsu1771*.

**Fig. 5:**
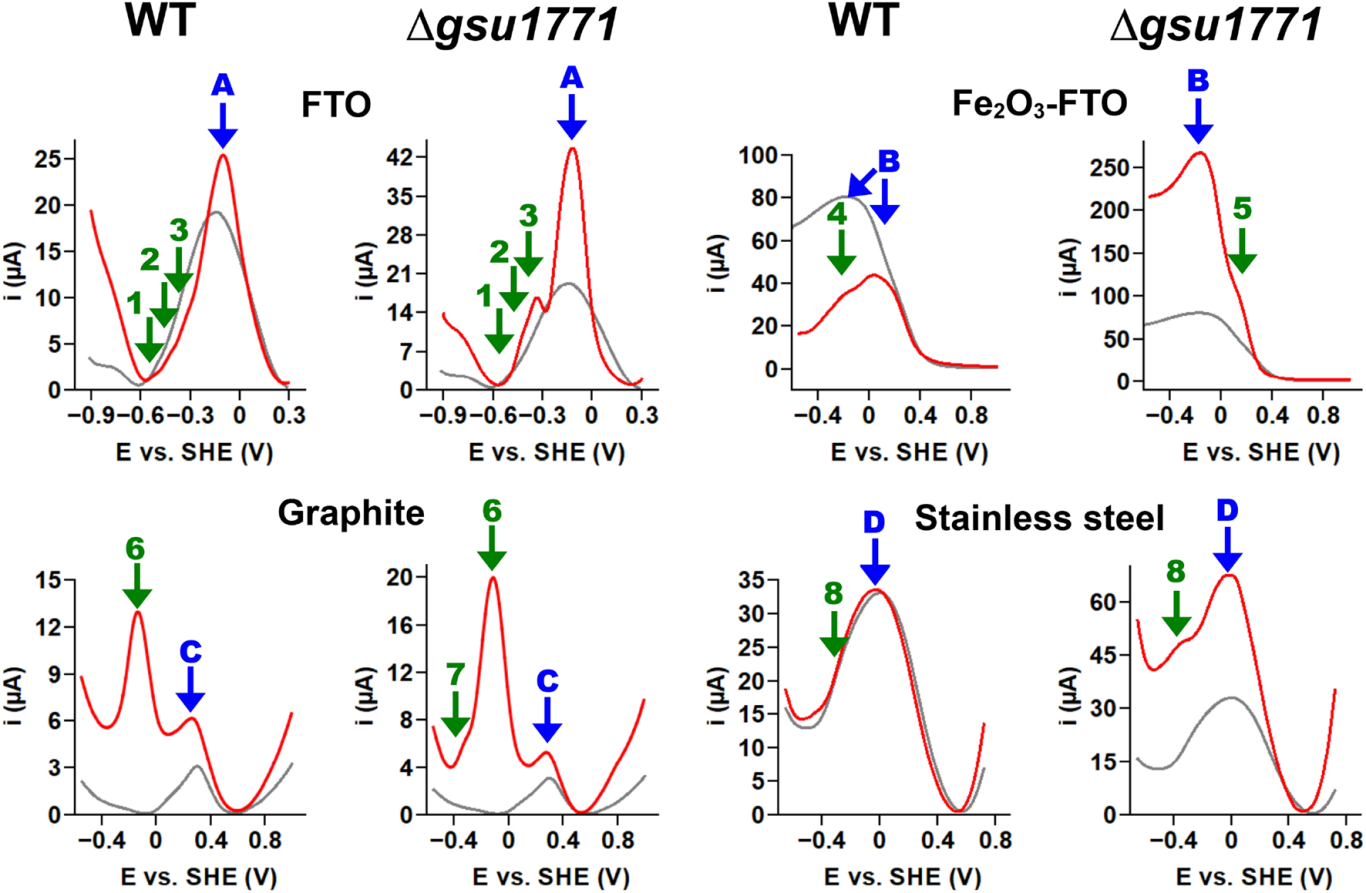
Square wave voltammetry (SWV) of *G. sulfurreducens* WT and Δ*gsu1771* biofilms on FTO (48 h), Fe_2_O_3_-FTO (48 h), graphite (96 h) and stainless steel (48 h). The gray lines represent the support material (without biofilms), and the red lines represent the electrochemical system after the addition of NaAc

SWV is an electrochemical technique capable of reducing the intrinsic capacitance of CV. The obtained voltammograms usually offer an excellent resolution of successive electroactive species in multicomponent systems like electroactive biofilms (Babauta and Beyenal 2015). Figure 5 shows the SWV responses of the biofilms in all the tested support materials in the presence of NaAc. This condition was chosen because the peak currents of the processes are better defined than in the absence of NaAc (data not shown). The WT biofilm in FTO shows a clear peak at − 0.12 V (peak A), corresponding to the bare FTO (see gray line); at more negative potentials, three small processes (inflections in the curve), absent in the bare FTO, appeared at − 0.33 V, − 0.42 V, and − 0.51 V (see arrows 1–3). In the Δ*gsu1771* biofilm, the process at − 0.33 V is better defined than in WT; additionally, the processes at − 0.42 V and − 0.51 V are also observed. The supplementary Fig. S3 shows a magnification of the SWV response to clarify the mentioned processes.

In Fe_2_O_3_-FTO, we observed the redox process of the Fe_2_O_3_ film; without biofilms, the process covers a wide potential region, but in WT, the process is more defined, and it appears around 0.12 V (Peak B). We are certain of the identity of this process due to a previous investigation in our work group, in which the process corresponding to peak 4 was also reported and attributed to the FTO current collector (Huerta-Miranda et al. 2023). The mutant strain presents a process around 0.15 V (peak 5) current higher than the WT response, suggesting that the mutant strain is transferring some of the electrons from acetate oxidation to the FTO current collector; unlike WT, in which the EET process causes only the reduction and dissolution of the Fe_2_O_3_ film. In the literature, there is evidence suggesting that the Fe_2_O_3_ films can enhance catalytic current production in electroactive biofilms in the presence of NaAc (Wen et al. 2022).

In the case of graphite, the process associated with the support material appears at 0.31 V (peak c); this process can be attributed to oxidation and reduction of some graphite surface functional groups (Soliman et al. 2016). We observed a process occurring around − 0.12 V in both strains (peak 6), which does not appear in the electrode without biofilm. The mutant strain presents a peak around − 0.33 V (peak 7), which does not appear in WT. Unlike CV, SWV allowed the observation of redox processes associated with *G. sulfurreducens* biofilms in this graphite electrode. Our research group is currently investigating why this specific graphite plate did not exhibit similar responses to those observed in previous reports (Huerta-Miranda et al. 2023). However, it is worth noting that the biofilms’ process at − 0.12 V agrees with our previous CV results in another type of graphite.

In stainless steel, the process associated with the support materials is found at 0.01 V (peak D). Another process of around − 0.33 V is observed in the biofilms of both strains (peak 8), which is absent on the bare electrode. This process is more evident in the mutant strain than in WT, and the fact that the current of the peak D increased compared to the support material without biofilm suggests that the electroactive capacity of the mutant biofilm allows the current to increase during acetate oxidation. The current increase in electrochemical responses of *G. sulfurreducens* has been studied by SWV alongside CV responses, and an increase in the peak current in turnover conditions for SWV corresponds to an increase in the limiting current in CV (Babauta and Beyenal 2017). Nevertheless, the reason why we observed an increase in the current in the presence of NaAc in SWV but not in CV must be further investigated. The kinetics of the electron transfer process in stainless steel differ from that on FTO, which could explain why we do not observe this phenomenon under the same analytic conditions.

### Gene expression analysis of selected genes

**Fig. 6:**
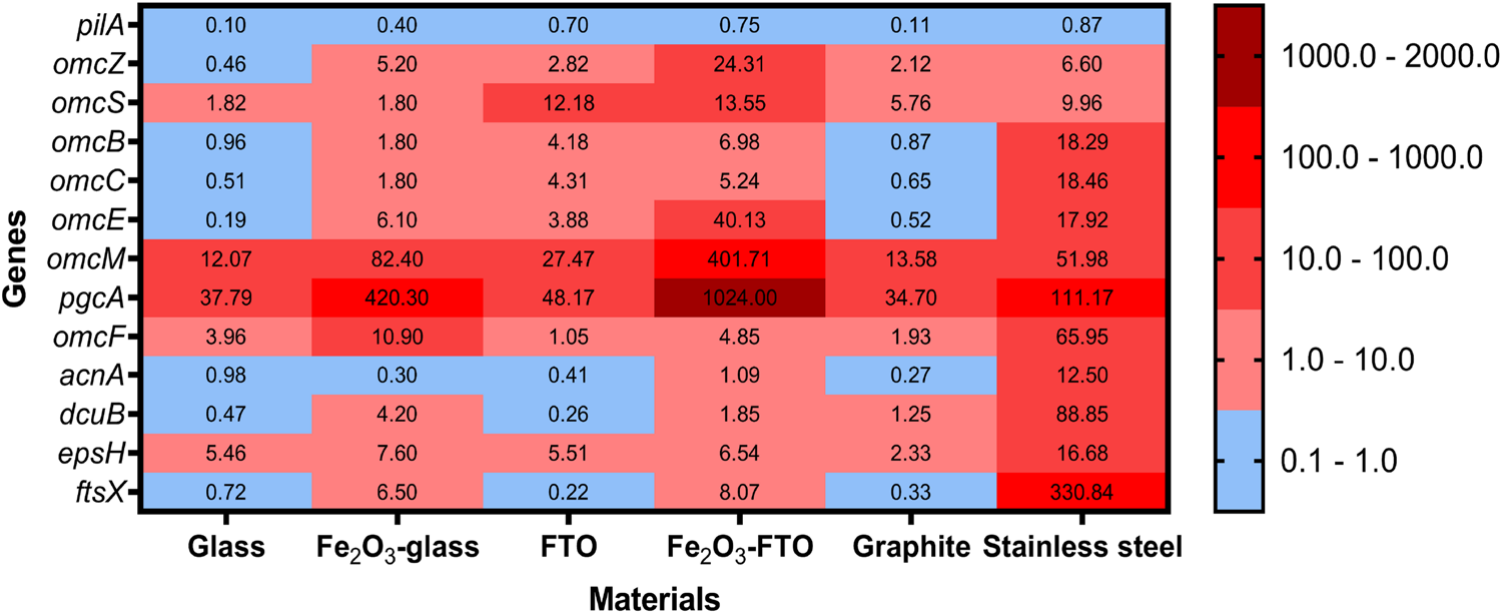
Heat map of the gene expression analysis of the *pilA*, *omcZ*, *omcS*, *omcB*, *omcC*, *omcE*, *omcM*, *omcF*, *pgcA*, *acnA*, *dcuB*, *epsH*, and *ftsX* genes in the Δ*gsu1771* biofilms compared to the WT biofilms. The biofilms were grown on different support materials at 48 h

With the aim of knowing more about the expression of some genes identified for their role in EET, Fig. 6 shows the gene expression of selected genes (*pilA*, *omcZ*, *omcS*, *omcB*, *omcC*, *omcE*, *omcM*, *omcF*, *pgcA*, *acnA*, *dcuB*, *epsH*, and *ftsX*) involved in EET and biofilm formation. Some of those genes were analyzed previously by our working group in the biofilm transcriptome of the Δ*gsu1771* mutant strain compared to the WT train. Both biofilms developed on glass supports, an inert and non-conductive material, thus avoiding the possibility that the electrode material was seen as an electron acceptor (Jaramillo-Rodríguez et al. 2023). In this work, in addition to glass, we also compared other support materials used for biofilm formation to study their influence not only on the structure of the biofilm but also on the expression of different *c-*type cytochromes, and other important components for metabolism and biofilm formation using RT-qPCR.

The *c-*type outer membrane cytochromes have been extensively studied in *G. sulfurreducens* for their role in EET (Ueki 2021). We found a higher expression of the following genes that encode *c-*type cytochromes in the Δ*gsu1771* biofilms compared to the WT biofilms on each of the materials used (Fig. 6): *omcF*, *omcM*, *omcS*, and *pgcA*. OmcF is a *c-*type monoheme outer membrane cytochrome that is required for Fe(III) reduction and current production on electrodes (Kim et al. 2005; Dantas et al. 2017); genetic studies show that OmcF has a key role in regulating genes encoding proteins necessary for Fe(III) reduction with OmcB (Kim et al. 2005) and electricity production in microbial fuel cells (OmcE and OmcS) (Kim et al. 2008). OmcM is a *c-*type tetraheme outer membrane cytochrome that is expressed during the Fe(III) and Pd(II) reduction (Aklujkar et al. 2013; Hernández-Eligio et al. 2020). OmcS is a *c-*type hexaheme outer membrane cytochrome essential for the reduction of insoluble Fe(III) and Mn(IV) oxides (Leang et al. 2010; Qian et al. 2011), and a recent report indicates that OmcS could form nanowires involved in long-range EET (Filman et al. 2019). PgcA is a *c-*type triheme extracellular cytochrome that facilitates the reduction of Fe(III) and Mn(IV) oxides (Zacharoff et al. 2017), which was the cytochrome with the highest expression of those selected in this work, which could be due that this cytochrome contributes to the reduction of several electron acceptors for its structural and biochemical characteristics (Fernandes et al. 2023), where it presents a greater expression in materials that contain Fe, which are stainless steel and hematite layers (Fig. 6).

Other *c-*type outer membrane cytochrome genes that were found differentially expressed on all materials were *omcB*, *omcC*, *omcE*, and *omcZ*. OmcB is a *c-*type dodecaheme outer membrane cytochrome involved in the reduction of soluble Fe(III) and which, together with two other proteins, forms a porin-cytochrome complex that transfers electrons across the electrode/biofilm interface (Leang et al. 2003; Liu et al. 2014). OmcC is a *c-*type dodecaheme outer membrane cytochrome homologous to OmcB, probably the result of genetic duplication (Leang and Lovley 2005). OmcE is a *c-*type tetraheme outer membrane cytochrome involved in the reduction of Fe(III) oxides (Mehta et al. 2005), and a recent report indicates that OmcE could form nanowires involved in long-distance EET (Wang et al. 2022a).

In this work, it is observed that *omcB*, *omcC*, and *omcE* are more expressed in materials covered by hematite, FTO, and stainless steel, but they are less expressed in glass and graphite in Δ*gsu1771* biofilms compared to WT biofilms. On the other hand, OmcZ is a *c-*type octaheme outer membrane cytochrome necessary for the transfer of electrons to electrodes and throughout the biofilm (Richter et al. 2009) and a recent report indicates that OmcZ could form nanowires essential for the formation of high current density biofilms that require long distance (Gu et al. 2023). In this work, it was found that *omcZ* is mostly expressed in conductive materials (Nevin et al. 2009; Franks et al. 2012) and covered by hematite, confirming that this cytochrome is necessary for the conductivity of biofilms grown on electrodes, but it was found that it is less expressed on glass, and this may be because it is a non-conductive material in Δ*gsu1771* biofilms compared to WT biofilms (Fig. 6).

In addition, the pili have also been extensively studied in *G. sulfurreducens* for their role in EET (Reguera et al. 2005; Reardon and Mueller 2013; Feliciano et al. 2015; Steidl et al. 2016). In this work, we found that there is a lower expression of the *pilA* gene (structural gene of the pili) in the Δ*gsu1771* biofilm compared to the WT biofilm on each of the materials used (Fig. 6).

On the other hand, the *epsH* gene belongs to the eps gene group, which controls the biosynthesis of extracellular polysaccharides in bacteria (Zhao et al. 2023). Through bioinformatic analysis, it was found that the *epsH* gene encodes a putative membrane protein that could be involved in the proteolysis (transpeptidation) of proteins with the signal peptide PEP-CTERM, similar to a sortase (Haft et al. 2006). In this way, it is identified that the *epsH* gene of *G. sulfurreducens* codes for a putative exopolysaccharide synthesis membrane protein H (exosortase). In this work, a higher expression of the *epsH* gene can be highlighted in the Δ*gsu1771* biofilm on all the materials, suggesting that this gene is related to the development of a thicker biofilm in Δ*gsu1771* compared to the WT strain (Fig. 6).

Another gene group that was found differentially expressed in biofilms of the Δ*gsu1771* biofilms are those involved in transport systems: *dcuB* and *ftsX*. The *dcuB* gene encodes a fumarate/succinate exchanger (C4 dicarboxylate transporter) (Butler et al. 2006), which allows the bacteria to take up fumarate and export succinate, making it essential for cell growth with fumarate as electron acceptor (Leang et al. 2009). In the analysis expression, we detected that *dcuB* is overexpressed in Δ*gsu1771* biofilms that grow on stainless steel, graphite, Fe_2_O_3_-glass, and Fe_2_O_3_-FTO (Fig. 6), which could indicate that the Δ*gsu1771* biofilms are consuming fumarate to be used as a final electron acceptor, in addition to these support materials, compared to the WT biofilms.

The *ftsX* gene encodes a cell division ABC transporter membrane protein FtsX (Schmidt et al. 2004). We detected that *ftsX* is overexpressed in the *Δgsu1771* biofilms that are grown on stainless steel, Fe_2_O_3_-glass, and Fe_2_O_3_-FTO (Fig. 6), which could indicate that greater cell division would be occurring and there would be more cells within these biofilms on these materials compared to those on the WT biofilms.

Furthermore, the *acnA* gene encodes an aconitase that catalyzes the reversible isomerization of citrate and isocitrate by cis-aconitate in the citric acid and glyoxylate cycles shown transcriptional changes (Gruer and Guest 1994). On glass, the Δ*gsu1771* biofilm does not change its expression but is overexpressed in Δ*gsu1771* biofilms grown on stainless steel and Fe_2_O_3_-FTO (Fig. 6), which could indicate a positive effect on the tricarboxylic acid metabolism of the Δ*gsu1771* biofilm in the presence of extracellular electron iron-based acceptors or donors, increasing its growth compared to the WT biofilm.

It is possible that the use of different conductive materials, especially those containing metals (FTO, hematite layers, and stainless steel), could be promoting the expression of some *c-*type cytochromes in Δ*gsu1771* biofilms compared to WT biofilms because these bacteria could evaluate the redox potential of the surfaces of each material and determine the precise EET pathway (Levar et al. 2014; Zacharoff et al. 2016; Joshi et al. 2021), which could be reflected in the different redox processes obtained by voltammetry of the biofilms grown on each support material. So *G. sulfurreducens* could reduce the Fe(III) contained in the hematite layers through outer membrane *c-*type cytochromes (Leang et al. 2003, 2010; Kim et al. 2005; Aklujkar et al. 2013; Zacharoff et al. 2017). And stainless steel contains several metals (iron, nickel, chromium, and molybdenum, among others) with which these bacteria could interact and favor their growth (Tang et al. 2019, 2021).

Table 3 shows the main characteristics of the biofilms developed on the different support electrodes; the influence of the chemical environment where *G. sulfurreducens* grows is more evident in the WT strain. It has been hypothesized that the long-range electron transfer in *G. sulfurreducens* could be explained by the combination of pili and associated cytochromes like OmcZ, OmcS, or OmcE because the recent cryo-electron microscopy studies have shown that nanowires in *G. sulfurreducens* are expressed differently in dependence of strains and the electron acceptor (Gralnick and Bond 2023).

**Table 3:** Influence of the chemical environment on the genotype, the phenotype and extracellular electron transfer

Support material	Important genes	EET response		Characteristics of biofilm	
		WT	Δ*gsu1771*	WT	Δ*gsu1771*
Conductive oxide (FTO)	*omcS*, *omcM*, *pgcA*	Current production due to metabolism	Enhanced electron transfer	Homogeneous and flat	Exhibited patterns related to column-like structures and channels
Non-conductive oxide (Fe_2_O_3_)	*omcS*, *omcZ*, *omcE*, *omcM*, *pgcA*	Biological degradation of the oxide	Enhanced biological degradation	Zones of high cell accumulation	Defined patterns, high columns
Carbon material (graphite)	*omcM*, *pgcA*	Responses with capacitive contributions	Low electron transfer	Heterogeneous distribution of biofilm	Defined patterns, wide channels
Electron donor (stainless steel)	*pgcA*, *ftsX*	Corrosion of the electrode	Enhanced electrode corrosion	Biofilms homogeneously distributed	Blurred patterns, high columns and cell accumulation

Additionally, elements at the inner membrane were found to be necessary for *G. sulfurreducens* to respire at determined potentials, regardless of the electron acceptor used (Levar et al. 2014; Zacharoff et al. 2016; Joshi et al. 2021). To date, it is unclear how the electron pathways switch and which other proteins are involved in each pathway. Nevertheless, our studies have contributed to the perspective on how *G. sulfurreducens* behave depending on the support electrodes. Other authors suggest that the modulation of the electrode potential may be another alternative to understanding the electron pathway selected (Levar et al. 2017; Howley et al. 2023). According to their results, the differences in the multi-heme cytochrome differential expression and the electrochemical data suggest that the potential modulation modifies the EET pathways and induces the expression of different genes depending on the growth conditions, as we did in this work.

## Conclusion

The use of different support materials to study *G. sulfurreducens* biofilms of two strains, wild type and *Δgsu1771*, allowed us to confirm the intrinsic characteristic of *Δgsu1771* for developing a thicker biofilm on all tested materials, both non-conductive and conductive. In addition, the overexpression of some genes (RT-qPCR results) that are involved in the extracellular electron transfer, such as *pgcA*, *omcS*, *omcM*, and *omcF*, as well as the overexpression of exopolysaccharides (*epsH*) was confirmed. Both strains presented different redox processes (voltammetry results) associated with each conductive material (FTO, Fe_2_O_3_-FTO, graphite, and stainless steel). Furthermore, we observed a substantial overexpression of *pgcA* and *omcF*, mainly in materials with Fe, suggesting some protein–metal interaction that these cytochromes could carry out. The results here open new perspectives for the study and application of *G. sulfurreducens* biofilms for developing hybrid biosystems like biosensors or bioanodes in BES. For the *Δgsu1771* mutant strain, our results show this mutant as a viable option for applications, taking advantage of its rapid extracellular electron transfer (EET) to final acceptors reflected by the high electric current that benefits bioelectrochemical processes required in the energy/environment and energy/health fields.

## Supplementary Information

Below is the link to the electronic supplementary material.

Supplementary file1 (DOCX 735 KB)

## Data Availability

Data will be made available on request.
