# Supplementary material for: Influence of support materials on the electroactive behavior, structure and gene expression of wild type and GSU1771-deficient mutant of Geobacter sulfurreducens biofilms
Source: Environ Sci Pollut Res Int. 2024 May 17;32(28):16740–59. doi: 10.1007/s11356-024-33612-3 (PMC12325454; doi:10.1007/s11356-024-33612-3)
Supplement: Supplementary file 1 — Supplementary file1 (DOCX 735 KB) [file 11356_2024_33612_MOESM1_ESM.docx]

**Supplementary information**

**Formation of electroactive biofilms on different support materials by two Geobacter sulfurreducens DL1 strains**

Luis Miguel Rodríguez-Torres^1,#^, Guillermo Antonio Huerta-Miranda^1,#^, Ana Luisa Martínez-García^2, 3^, Dalia Alejandra Mazón-Montijo^2, 3, 4^, Alberto Hernández-Eligio^1, 4^, Margarita Miranda-Hernández^5^, Katy Juárez^1^*

^1^ Departamento de Ingeniería Celular y Biocatálisis, Instituto de Biotecnología, Universidad Nacional Autónoma de México, Av. Universidad 2001. Col. Chamilpa, 62210 Cuernavaca, Morelos, México

^2^ Centro de Investigación en Materiales Avanzados S. C., subsede Monterrey, Grupo de Investigación DORA-Lab, 66628, Apodaca, N. L., México.

^3^ Tecnológico Nacional de México campus Nuevo León (TECNL), Centro de Investigación e Innovación Tecnológica (CIIT), Grupo de Investigación DORA-Lab, 66629, Apodaca, N. L., México.

^4^ Investigadores por México, CONAHCYT, Ciudad de México, México.

^5^ Instituto de Energías Renovables, Universidad Nacional Autónoma de México, Priv. Xochicalco, 62580 Temixco, Morelos, México

^#^ These authors contributed equally as first author

**Corresponding author:* [*katy.juarez@ibt.unam.mx*](about:blank)

**Table S1. Primer sequences used for RT-qPCR validations**

| Oligonucleotides | Description | Reference/source |
| --- | --- | --- |
| qPCRacnAFw | CCCGCTACAACTACCACTCC | Andrade *et al*., 2021 |
| qPCRacnARev | CGCAAAAGGTTTTCCAGAAG | Andrade *et al*., 2021 |
| qPCRdcuBFw | GCCATCAAGACCGGTATCC | Andrade *et al*., 2021 |
| qpCRdcuBrev | AGCCGAGGAAAAAGGTCACT | Andrade *et al*., 2021 |
| qPCRepsHfw | CTATGCAGCCCTCACCATCC | Jaramillo-Rodríguez *et al.*, 2023 |
| qPCRepsHrv | GTGAATGACATTGCCCTCGC | Jaramillo-Rodríguez *et al.*, 2023 |
| qPCRftsXfw | AGCGGCAAGATCCAGGTTAC | This study |
| qPCRftsXrv | TAGGAAACGCGTGAAGTCCC | This study |
| qPCRomcBfw | GGAGTATGTGGCATCCCTTG | Hernandez-Eligio *et al*., 2020 |
| qPCRomcBrev | ACCGTTGGCATTCGTATCTC | Hernandez-Eligio *et al*., 2020 |
| qPCRomcCfw | AGAGTACGTGGCATCCCTTG | Hernandez-Eligio *et al*., 2020 |
| qPCRomcCrev | CCGTTGGCATTCGTATCTCT | Hernandez-Eligio *et al*., 2020 |
| qPCRomcEfw | CCAGATCTGCGTGTTCTGTC | Hernandez-Eligio *et al*., 2020 |
| qPCRomcErev | CATGCTGCTGGACGAGTAGA | Hernandez-Eligio *et al*., 2020 |
| qPCRomcFfw | GACGTGGCGGCCTACATC | This study |
| qPCRomcFrv | CGACGTACTCCCCGATCTTC | This study |
| qPCRomcMfw | TGGAGACTACCCATGCTGAA | Hernandez-Eligio *et al*., 2020 |
| qPCRomcMrev | AGACGTCGAGGTGCTCGTAT | Hernandez-Eligio *et al*., 2020 |
| qPCRomcSfw | TCCTACCAGAACAGCAACGA | Hernandez-Eligio *et al*., 2020 |
| qPCRomcSrev | ATAGGAACCGCTCAGGGACT | Hernandez-Eligio *et al*., 2020 |
| qPCRomcZfw | AAGCCGACTGTCTCGAGTGT | Hernandez-Eligio *et al*., 2020 |
| qPCRomcZrev | CGGAGGTATTGATGCAGCTT | Hernandez-Eligio *et al*., 2020 |
| qPCRpilAfw | TCGGTATTCTCGCTGCAAT | Andrade *et al*., 2021 |
| qPCRpilArev | AATGCGGACTCAAGAGCAGT | Andrade *et al*., 2021 |
| qPCRpgcAFw | GTCTCCAGAGTGCCGTAAGC | Hernandez-Eligio *et al*., 2022 |
| qPCRpgcARev | AGTAGGAGCCACTGCCAAGA | Hernandez-Eligio *et al*., 2022 |

**References**

Andrade A, Hernández-Eligio A, Tirado AL, Vega-Alvarado L, Olvera M et al (2021) Specialization of the Reiterated Copies of the Heterodimeric Integration Host Factor Genes in *Geobacter sulfurreducens*. Front Microbiol 12:626443. https://doi.org/10.3389/fmicb.2021.626443

Jaramillo-Rodríguez JB, Vega-Alvarado L, Rodríguez-Torres LM, Huerta-Miranda GA, Hernández-Eligio et al (2023) Global transcriptional analysis of *Geobacter sulfurreducens gsu1771* mutant biofilm grown on two different support structures. bioRxiv, Preprint posted March 01. https://doi.org/10.1101/2023.02.27.530372

Hernández-Eligio A, Huerta-Miranda GA, Martínez-Bahena S, Castrejón-López D, Miranda-Hernández M et al (2022) GSU1771 regulates extracellular electron transfer and electroactive biofilm formation in *Geobacter sulfurreducens*: Genetic and electrochemical characterization. Bioelectrochemistry 145:108101. https://doi.org/10.1016/j.bioelechem.2022.108101

Hernández-Eligio A, Pat-Espadas AM, Vega-Alvarado L, Huerta-Amparán M, Cervantes FJ et al (2020) Global transcriptional analysis of *Geobacter sulfurreducens* under palladium reducing conditions reveals new key cytochromes involved. Appl Microbiol Biotechnol 104:4059-4069. <https://doi.org/10.1007/s00253-020-10502-5>


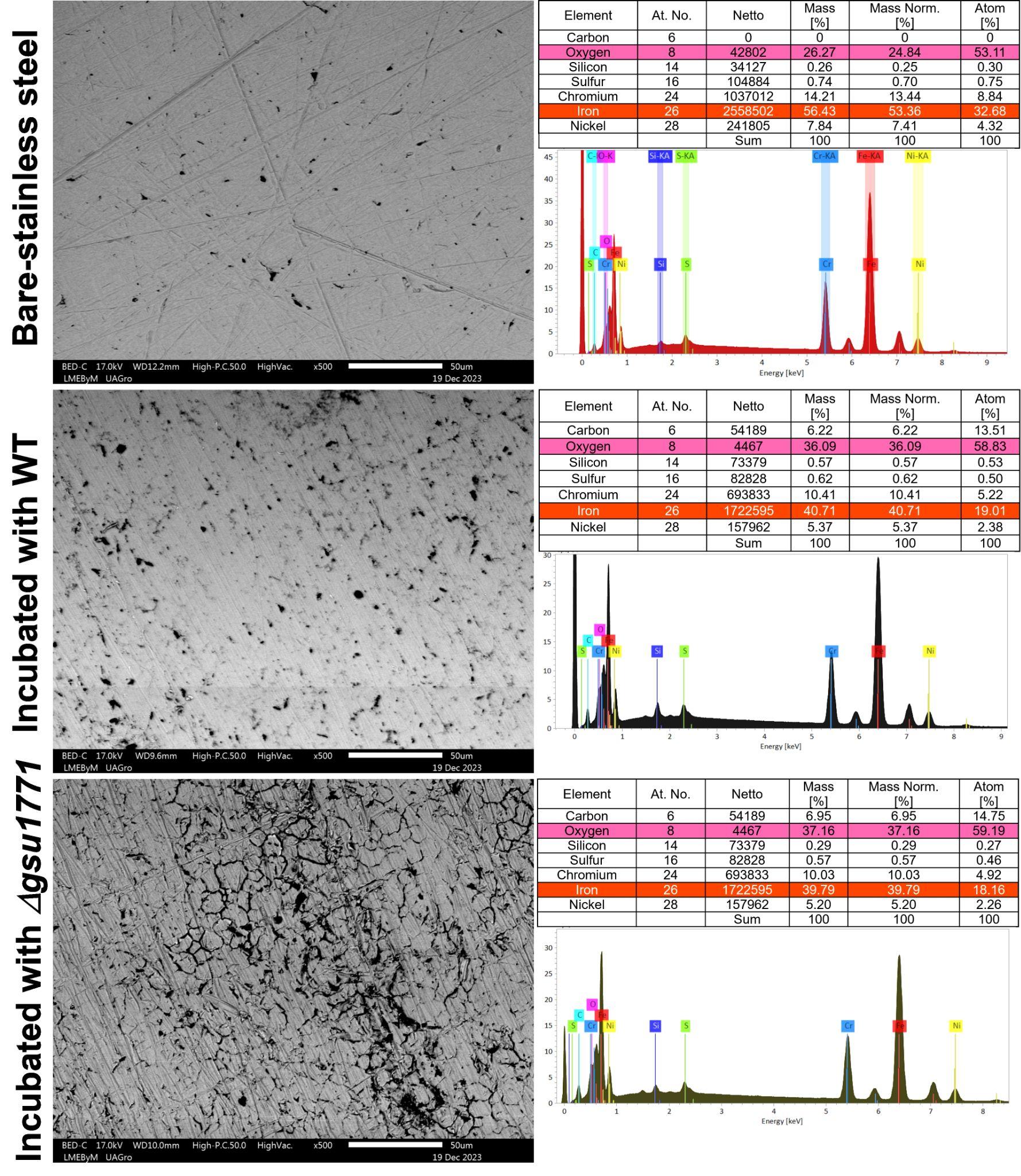


**Fig. S1** SEM images and EDX spectrum of the bare-stainless steel electrode without any treatment and the electrodes after 40 days of incubation with the *G. sulfurreducens* strains.


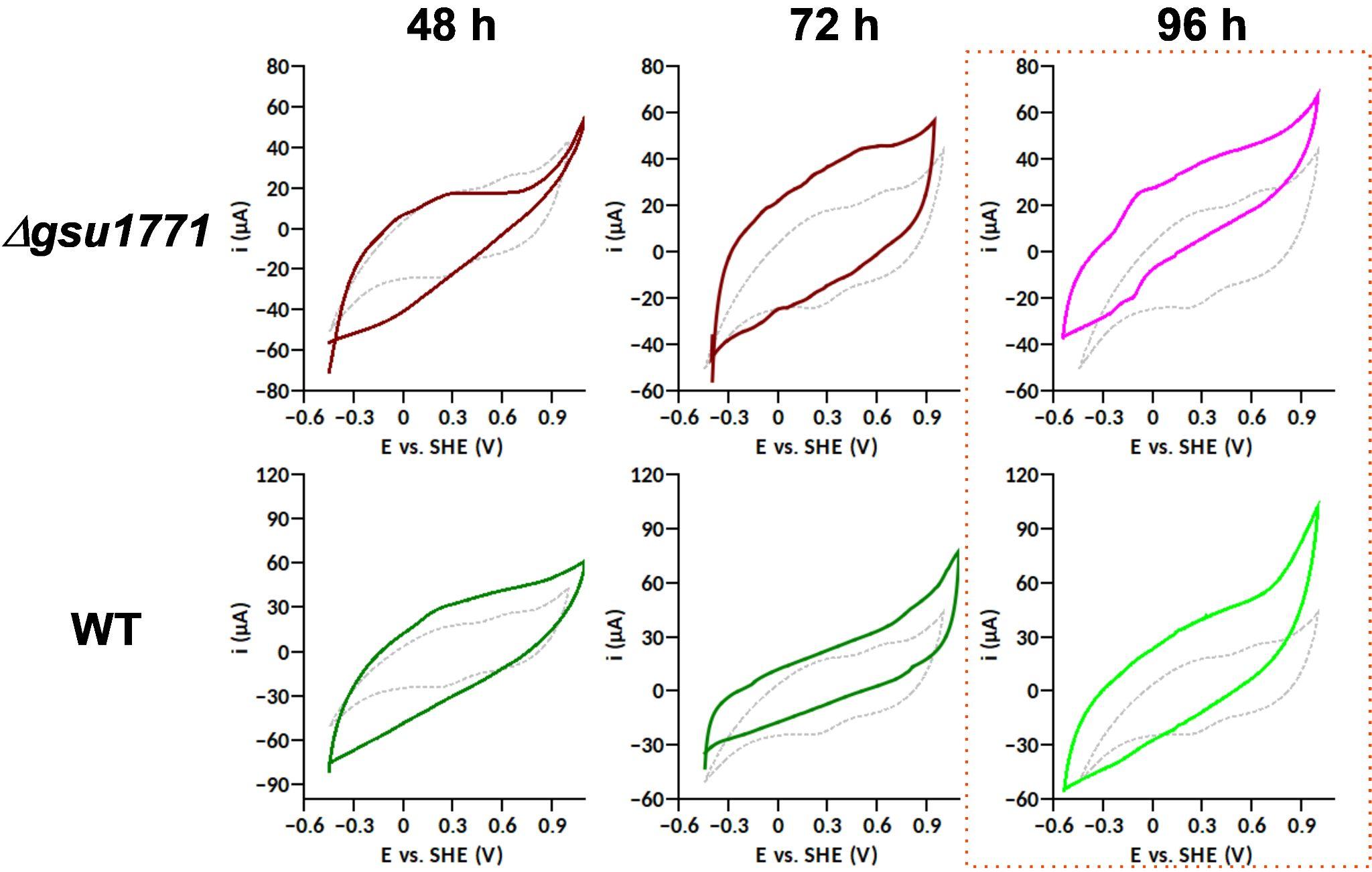


**Fig. S2** Cyclic Voltammetry (CV) of *G. sulfurreducens* WT and Δ*gsu1771* biofilms on graphite at different incubation times in the basal medium as the electrolyte at 0.01 V/s scan rate. The gray dashed lines represent the support material (without biofilms).


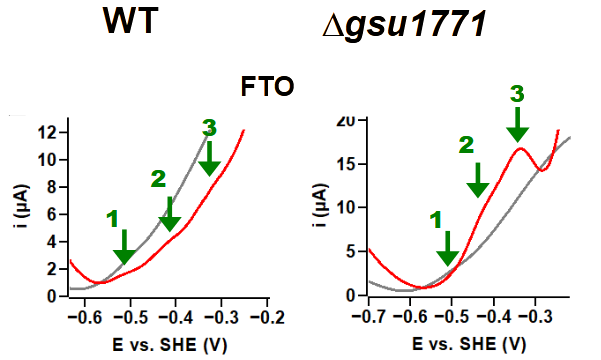


**Fig. S3** Magnification of the Square Wave Voltammetry (SWV) response of *G. sulfurreducens* WT and Δ*gsu1771* biofilms on FTO (48 h) at 0.001 V/s and 0.01 V amplitude. The gray line represents the electrode material (without biofilms).
